# BALL - biochemical algorithms library 1.3

**DOI:** 10.1186/1471-2105-11-531

**Published:** 2010-10-25

**Authors:** Andreas Hildebrandt, Anna Katharina Dehof, Alexander Rurainski, Andreas Bertsch, Marcel Schumann, Nora C Toussaint, Andreas Moll, Daniel Stöckel, Stefan Nickels, Sabine C Mueller, Hans-Peter Lenhof, Oliver Kohlbacher

**Affiliations:** 1 Center for Bioinformatics Saar, Saarland University, Saarbrücken, Germany; 2 Center for Bioinformatics Tübingen, Eberhard-Karls-Universität Tübingen, Germany; 3 Intel Visual Computing Institute of Saarland University, Germany

## Abstract

**Background:**

The Biochemical Algorithms Library (BALL) is a comprehensive rapid application development framework for structural bioinformatics. It provides an extensive C++ class library of data structures and algorithms for molecular modeling and structural bioinformatics. Using BALL as a programming toolbox does not only allow to greatly reduce application development times but also helps in ensuring stability and correctness by avoiding the error-prone reimplementation of complex algorithms and replacing them with calls into the library that has been well-tested by a large number of developers. In the ten years since its original publication, BALL has seen a substantial increase in functionality and numerous other improvements.

**Results:**

Here, we discuss BALL's current functionality and highlight the key additions and improvements: support for additional file formats, molecular edit-functionality, new molecular mechanics force fields, novel energy minimization techniques, docking algorithms, and support for cheminformatics.

**Conclusions:**

BALL is available for all major operating systems, including Linux, Windows, and MacOS X. It is available free of charge under the Lesser GNU Public License (LPGL). Parts of the code are distributed under the GNU Public License (GPL). BALL is available as source code and binary packages from the project web site at http://www.ball-project.org. Recently, it has been accepted into the debian project; integration into further distributions is currently pursued.

## Background

Developing programs for structural bioinformatics is a difficult and often tedious task. Even if the algorithms have been carefully designed, the programmer has to solve a variety of complex and recurring problems not fundamentally related to the algorithm at hand, but necessary for real-world applications. Not only more advanced tasks like inferring missing atoms or bonds, energy evaluations, or structural minimization require considerable programming effort that can hardly be repeated for every new project, but also the most basic and mundane steps. For example, many molecular file formats are as hard to parse correctly as they are to write. To avoid costly and error-prone re-inventing of the wheel for any new structural bioinformatics application, two approaches can be imagined: a collection of loosely coupled tools and utilities for recurring subtasks, or powerful libraries and frameworks for rapid application development (*RAD*). Obviously, the second approach encompasses the first, i.e., creating small, specialized tools for a pipeline concept is trivial when relying on such a library. In addition, it allows its users simple access to the molecular data structures and algorithms that form building blocks of many algorithmic approaches and that often require complex implementations. With the Biochemical Algorithms Library (BALL) [1], we have created a versatile C++ class library for structural bioinformatics that is supplemented with a Python interface for scripting functionality and a number of applications like the molecular modeling frontend BALLView [2]. BALL has been used successfully for a large number of projects, both of our own (e.g. [3-7]) and of external researchers (for a small selection of recent publications, see e.g. [8-14]). In recent years, BALL has seen a significant increase in functionality and substantial useability improvements. It has been ported to further operating systems; indeed, it currently supports all major brands. Moreover, BALL has evolved from a commercial product into a free-of-charge, open source software licensed under the Lesser GNU Public License (LGPL).

**Figure 1:**
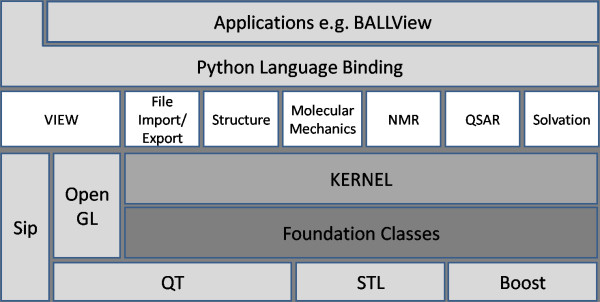
**Overview of the structure of BALL**. The diagram shows the general layout of the structure of BALL, where every box symbolizes one library or fundamental layer.

Several frameworks for structural bioinformatics have been reported in the literature; most of them, however, with a different focus, scope, or intended audience. Hence, comparison with these projects is difficult in general. Among those projects, the most similar in scope and functionality are commercial packages like the suites from Schrödinger [15] or the Chemical Computing Group's Molecular Operating Environment (MOE) [16]. While these packages typically focus on sophisticated graphical user interfaces for concrete modelling tasks, they also provide powerful scripting interfaces aimed at developers. Notable open source projects in the field include Biopython [17], PyMOL [18] (which provides extensive scripting functionality apart from the molecular viewer), CDK [19], MESHI [20], JOELib [21], the EGAD Library [22], and StrBioLib [23]. To the best of our knowledge, BALL offers the widest range of functionality for rapidly and robustly developing applications in structural bioinformatics, it is growing fast and can be easily extended. It addresses users of the implemented techniques as well as designers of completely new approaches. An overview of BALL's design can be found in Figure 1.

A full description of BALL's functionality would fall well outside of the scope of this note; the current version (1.3 at the time of writing) contains more than 730 classes and more than 700,000 lines of code (a comprehensive overview can be found in the online documentation at http://www.ball-project.org). Instead, we want to briefly point out some of BALL's most important features, particularly highlighting those that have been added since [1]. In addition, we will show some ways in which the use of such an *RAD *framework can simplify the life of scientists and developers.

## Implementation

BALL has been implemented in C++, with some extensions written in Python.

## Results

From the start of its development in 1996, BALL's design principles have been wide functionality, ease of use, openness, and robustness.

### Wide functionality

To demonstrate BALL's rich functionality, we describe a typical application - namely docking - and point out how BALL simplifies its implementation. In this case, a large amount of time and code is devoted to the problem of importing the docking partners into suitable structural representations and preparing them for further use. Typically, this preparation consists in reading the molecules from diverse file formats, checking their content, adding missing information, running the actual docking algorithm, and, finally, analysing its result.

Reading the data doubtlessly belongs to the most recurring tasks in molecular software development. BALL supports a rich variety of molecular structure formats. While the previously published version [1] only supported the molecular file formats PDB and MOL2, version 1.3 additionally reads and writes MOL, HIN, XYZ, KCF, and SD files. Besides molecular files, it also supports a variety of other data sources, like DCD, DSN6, GAMESS, JCAMP, SCWRL, and TRR. Export to most of these formats is possible as well. Apart from importing molecules from external sources, the new version also offers rich functionality for generating and editing molecules. For instance, given only the amino acid sequence and the corresponding torsion angles, BALL's PeptideBuilder creates a 3D structure of that peptide. More general molecular structures, e.g., of ligands, can be generated from SMILES-expressions or programmatically by explicitly inserting atoms and bonds.

The next step - not only in docking but in all applications processing molecular structures - is to validate the structures and to prepare the data for the following tasks. Some atoms, in particular, hydrogens, are often missing, and structural information such as connectivity or bond orders are often incorrect or even missing. For proteins, DNA, and RNA, BALL can automatically infer much of the missing information from an extensible fragment database. This can also be used for validating given structures. A rotamer library allows the user to easily determine a protein's most likely side-chain conformations or to easily switch between several rotameric states. Both, fragment database and rotamer library have been significantly improved since BALL's first publication. Other molecules with a more diverse chemistry, such as ligands, require more sophisticated approaches to infer missing structural information. BALL's new BondOrderAssigner[5] heuristically determines all possible bond order assignments for a given ligand sorted by their probability. Favorable 3D conformations can be achieved by employing BALL's new QuickOptimizer, a randomized MDSimulator/Minimizer in combination with several force fields (see below). Also among the new features are a kekulizer and an aromaticity processor.

Once the input has been prepared, the two core problems in protein docking are the generation of docking poses and their evaluation. For both tasks, BALL offers rich functionality. The first is facilitated by the preparation functionality (as described above) and BALL's transformation processors. Here, BALL's selection mechanisms also simplify matters by allowing, for instance, the selection of certain parts of the molecule through easily formulated expressions. Via a selector class, certain predicates like element type can be used as a selection filter. Additionally, BALL now provides an expression class which allows selecting subsets of objects by given SMILES and SMARTS strings as well as by BALL predicates.

The second task often amounts to energy evaluations and/or the checking of certain criteria. The former version of BALL provided the force field classes CHARMM [24] and AMBER [25]. In the current version, an implementation of the Merck Molecular Force Field (MMFF94) [26], a fully parameterized force field that allows handling of virtually all kinds of organic molecules, has been added as well as some non-differentiable scoring functions.

Force fields can not only be used for scoring: BALL's minimizer and molecular dynamics classes can be combined with all of the implemented force fields. Minimizers and simulation classes also support selection, allowing the user to freely specify a set of movable atoms from all atoms used for force field computation. This is useful in a variety of contexts, e.g. when estimated hydrogen positions have to be reoptimized. Since the last version, we have greatly extended the minimization capabilities [27], now offering standard (steepest descent, conjugate gradient) and the best currently known methods (L-BFGS and shifted L-VMM).

In addition to the features described above, version 1.3 has been greatly extended with further functionality. For instance, secondary structure prediction and hydrogen-bond detection [28] are now available.

In summary, BALL has developed into a powerful tool for *RAD *covering fundamental functionality as well as complex applications like molecular docking and drug design. Due to its modular architecture, all classes and algorithms can be combined in a building block manner to easily implement even complex methods.

### Ease of use

One measure of the usefulness of an *RAD *platform is the time it saves compared to developing the functionality from scratch. Hence, ease of use and a shallow learning curve are important goals for any large-scale framework. On the other hand, after some time of familiarization with the library, users will usually want to fine-tune the methods, choose detailed parameters, or even exchange parts of the algorithms. Supporting these advanced users bears the risk of conflicting with the ease-of-use principle, for instance, by confusing the user with a wide array of tuneable options. BALL has been very carefully designed to address both groups of users, experts and novices alike, simultaneously. For example, most algorithms implemented in BALL accept a wide range of options to fine-tune their behavior, but all of these come with sensible defaults. In this way, a novice user can just instantiate a class and use it successfully, while experts can adapt the options to their individual needs. Similarly, the versatile Python interface offered by BALL appeals to both groups of users, albeit in different ways: while novice users and non-programming experts can profit from the easy-to-learn scripting languages, experts can use it to create powerful scripts.

SIP is used to automatically create python classes for all relevant C++ classes to allow for the same class interfaces. The Python class has the same name as the C++ class, so porting code that uses BALL from C++ to Python (and vice versa) is usually a trivial task. For instance, the following C++ code

// *read a PDB file*

PDBFile file (" test . pdb");

System S;

file >> S;

file . close ( );

// *add missing information*

// *e . g . hydrogens and bonds*

FragmentDB fragment_db (" ");

S . apply (fragment_db . normalize_names);

S . apply (fragment_db . add_hydrogens);

S . apply (fragment_db . build_bonds);

// *check for charges, bond lengths *,

// *and missing atoms*

ResidueChecker checker (fragment_db);

S . apply (checker);

// *create an AMBER force field*

AmberFF FF;

S . deselect ( );

FF . setup (S);

Selector selector (" element (H) ");

S . apply (selector);

// *optimize the hydrogen ' s positions*

ConjugateGradientMinimizer minimizer;

minimizer . setup (FF);

minimizer . setEnergyOutputFrequency (1);

minimizer . minimize (50);

// *write a PDB File*

file . open (" test_out . pdb", ios : : out);

file << S;

file . close ( );

translates to

# *read a PDB file*

file = PDBFile (" test . pdb")

system = System ( )

file . read (system)

file . close ( )

# *add missing information*

# *e . g . hydrogens and bonds*

Fragment_db = FragmentDB(" ")

system . apply (fragment_db . normalize_names)

system . apply (fragment_db . add_hydrogens)

system . apply (fragment_db . build_bonds)

# *check for charges, bond lengths *,

# *and missing atoms*

checker = ResidueChecker (fragment_db)

system . apply (checker)

# *create an AMBER force field*

FF = AmberFF( )

system . deselect ( )

FF . setup (system)

selector = Selector (" element (H) ")

system . apply (selector)

# *optimize the hydrogen ' s positions*

minimizer = ConjugateGradientMinimizer ( )

minimizer . setup (FF)

minimizer . setEnergyOutputFrequency (1)

minimizer . minimize (50)

# *write a PDB File*

outfile = PDBFile (" test_out . pdb", File .MODE_OUT)

outfile . write (system).

outfile . close ( )

Since the Python interface is fully integrated into the molecular viewer and modeling tool BALLView [2], the effects of the scripts can be visualized directly. Also, the interface provides a simple way to automatize BALLView's behaviour.

Finally, a number of tutorials guide inexperienced users through the writing of their first applications.

These tutorials are provided with BALL's extensive documentation and have recently been supplemented with a code library for recurring tasks on our wiki http://ball-trac.bioinf.uni-sb.de/wiki.

### Robustness

Apart from substantially simplifying the creation of applications, the use of *RAD *frameworks can also help greatly in ensuring their correctness and improving their robustness, since the code in the library has often been used and tested in a variety of situations by a large number of people. To improve robustness, BALL employs a large number of regression tests that are regularly executed on a number of different platforms. In this way, it is easy to determine whether a change in some part of the code will lead to a regression in another part, or whether a new compiler release, for instance, will result in different behavior.

## Conclusions

The Biochemical Algorithms Library BALL is a comprehensive rapid application development framework for structural bioinformatics. BALL has been carefully designed to address programming experts as well as novices. Users can take advantage of BALL's rich functionality being offered an extensive framework of data structures and algorithms through both, C++ and the python scripting interface. A variety of standard structural bioinformatics algorithms are offered and new algorithms can be easily added.

With the new release 1.3 BALL is complemented with a number of key features, e.g. additional file formats, molecular edit-functionality, and new molecular mechanics force fields. Fundamental parts of BALL's core have been rewritten, and the build system was switched to CMake to increase portability.

## Availability and Requirements

*Project name*: BALL - Biochemical Algorithms Library

*Project home page*: http://www.ball-project.org

*Operating systems*: Linux, Windows, and MacOS X

*Programming language*: C++, python

*License*: Lesser GNU Public License (LGPL)

*Restrictions to use by non-academics*: None

## Authors' contributions

AH, HPL, and OK are heading the project. All authors contributed significantly to the project through programming, documenting, and testing. All authors read and approved the final manuscript.
